# Large Cholesterol Granuloma of the Middle Ear Eroding into the Middle Cranial Fossa

**DOI:** 10.1155/2017/4793786

**Published:** 2017-06-22

**Authors:** Tessei Kuruma, Tohru Tanigawa, Yasue Uchida, Ogawa Tetsuya, Hiromi Ueda

**Affiliations:** Department of Otorhinolaryngology, Head and Neck Surgery, Aichi Medical University, Aichi, Japan

## Abstract

**Background:**

Cholesterol granuloma of the middle ear is extremely rare in comparison to cholesterol granuloma of the petrous apex but sometimes shows an aggressive course.

**Case Report:**

We report herein a case involving a large, aggressive cholesterol granuloma of the middle ear that eroded the middle cranial fossa. A 64-year-old woman presented with pain in the left ear and hearing loss. Cholesterol granuloma was finally diagnosed from diffusion-weighted imaging, and cortical mastoidectomy was performed with canal wall down tympanoplasty type III. Recovery was uneventful recovery and the patient well at the 3-year follow-up.

**Conclusion:**

This case demonstrates the rare but clinically important pathology of aggressive cholesterol granuloma of the middle ear.

## 1. Introduction

Cholesterol granuloma is thought to be caused by a foreign body reaction to cholesterol crystals released by the breakdown of blood and local tissue. Cholesterol is an unusual endogenous substance; in that it is relatively resistant to absorption by giant cells [1].

Cholesterol granulomas are predominantly found incidentally during surgical treatment of cholesteatomas and chronic otitis media and can also occur in the context of posttraumatic hemorrhage of the temporal bones [2]. In each case, internal hemorrhage is thought to be the cause of the formation of cholesterol granuloma.

Cholesterol granuloma may erode into the middle ear, mastoid bone, or petrous apex. Cholesterol granuloma at the petrous apex presents with symptoms related to bony erosion (e.g., sensorineural hearing loss, tinnitus, vertigo, or cranial nerve impairment) [3]. However, aggressive and destructive cholesterol granulomas of the middle ear are extremely rare [4]. The present report describes a case of huge cholesterol granuloma extending to the middle cranial fossa.

## 2. Case Presentation

**Figure 1:**
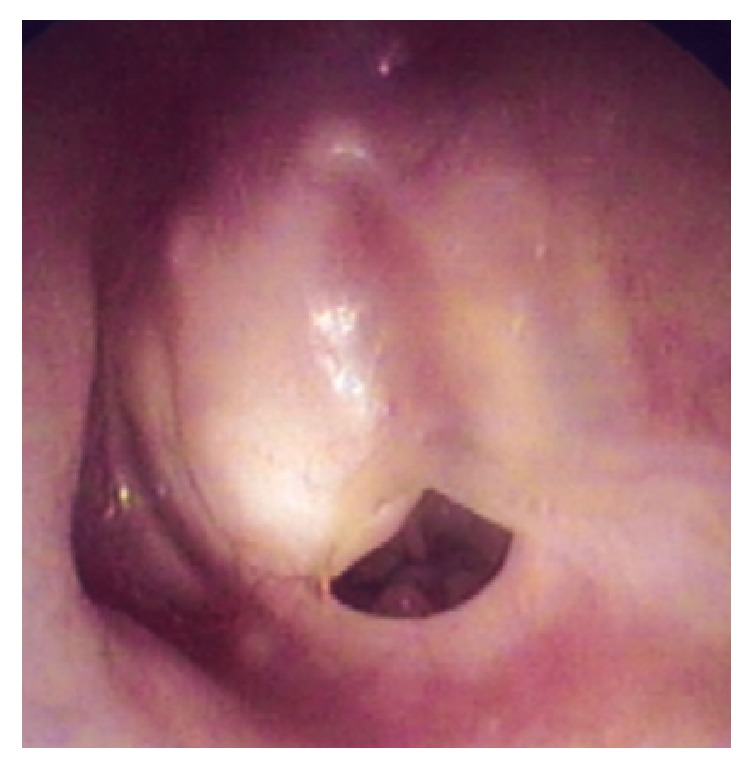
The left tympanic membrane is thick and white. A left tympanic membrane perforation is evident, but with no discharge.

**Figure 2:**
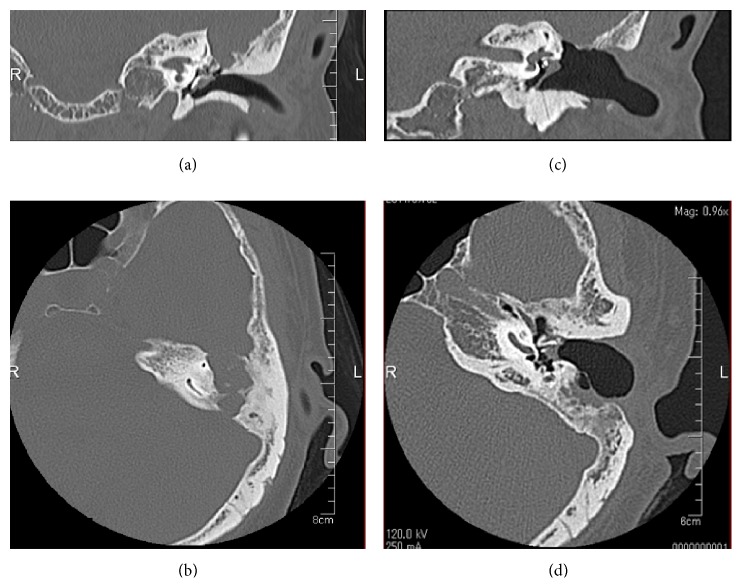
(a) Coronal CT of the temporal bone showing bony destruction of the middle cranial fossa. (b) Axial CT showing bony destruction of the middle and posterior cranial fossae. (c, d) Postoperative CT at 3 years after the operation shows no recurrence of cholesterol granuloma.

**Figure 3:**
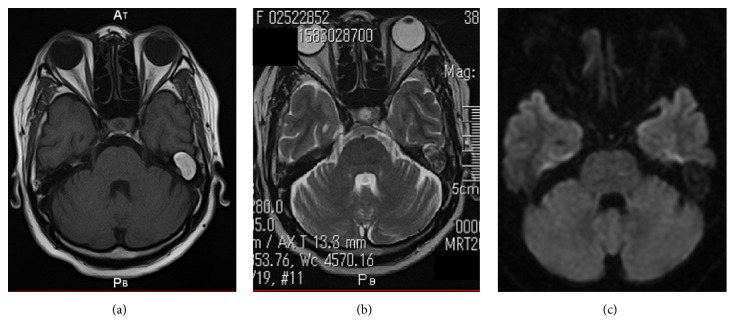
(a) Axial T1-weighted temporal bone MRI showing a signal-hyperintense mass neighboring the posterior cranial fossa and extending to the middle fossa. (b) Axial T2-weighted imaging of the temporal bone shows a high-intensity periphery and low-intensity central mass. (c) DWI shows an isointense signal rather than high intensity.

A 64-year-old woman presented to our outpatient department with a 2-year history of otalgia and hearing loss in the left ear. She had a history of recurrent otitis media in childhood but had not experienced otorrhea in adulthood. The left tympanic membrane was thick and white on otomicroscopic examination, with no discharge from an obvious perforation of the left tympanic membrane (Figure 1). Audiologic testing revealed a left-sided mixed hearing loss of 56.8 dB pure tone average (PTA). Computed tomography (CT) showed a soft-tissue mass that had destroyed the bony plate of the posterior and middle cranial fossa (Figures 2(a) and 2(b)). On magnetic resonance imaging (MRI), the mass displayed signal hyperintensity on T1-weighted imaging (Figure 3(a)) and a high-intensity periphery surrounding a low-intensity central signal on T2-weighted imaging (Figure 3(b)). The mass compressed the middle cranial fossa without invasion of the brain. Diffusion-weighted imaging (DWI) revealed an isointense signal, rather than the high intensity that would typically be seen with cholesteatoma (Figure 3(c)). Based on these findings, cholesterol granuloma was diagnosed.

The operation was performed using a retroauricular approach. The mastoid portion was found to be filled with a dark purple tumor that was thought to represent cholesterol granuloma. The cyst wall of the cholesterol granuloma was very thick. We removed the tumor by dissecting the temporal bone from the mastoid to the epitympanum. We removed the granulation tissue in the epitympanum. A sclerotic lesion was found around the ossicles, and the pathway from the epitympanum to the aditus was obstructed by thick bone

**Figure 4:**
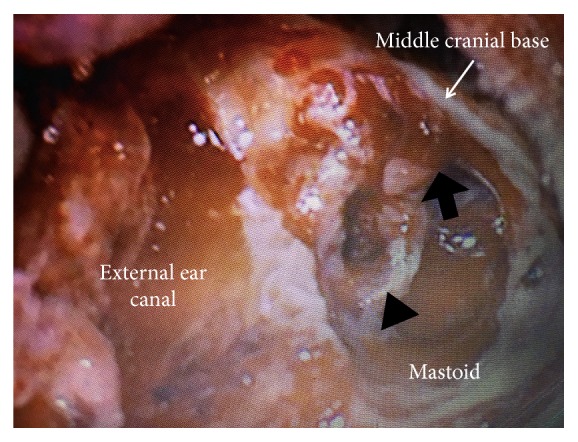
Intraoperative picture showing pulsating bleeding was seen from the dura in the middle cranial fossa (arrow) in removing the cholesterol granuloma (arrowhead) from the dura. Black arrow indicates the dura in the middle cranial fossa.

When we removed the cholesterol granuloma under the middle cranial fossa that had invaded into the cranium, pulsatile bleeding was seen. This was attributed to a blood vessel of the dura in the middle cranial fossa (Figure 4). The bleeding was stopped through the use of a coagulating device.

**Figure 5:**
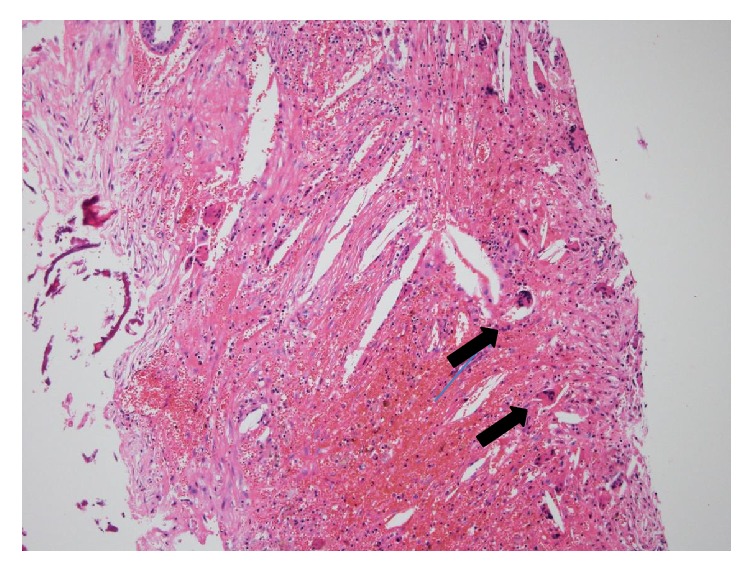
The pathological specimen showing a typical cholesterol granuloma. Granulation contains clefts of cholesterol crystals, giant cells, and infiltration of inflammation tissue (arrow). HE stain, 100x.

The solid component of the cholesterol granuloma was removed from the dura. Cerebrospinal fluid (CSF) flow was noted from a defect in the dura in the middle cranial fossa and was terminated by applying pressure gauze. We covered the defect of the dura in the middle cranial fossa using connective tissue. We did not use bone, because we expected that the scar tissue would expand and cover the defect in the dura. We performed canal wall down tympanoplasty type III (Wullstein classification). Histologically, the lesion was consistent with a typical cholesterol granuloma; the granulation tissue contained clefts of cholesterol crystals, giant cells, and infiltration with inflammatory cells (Figure 5).

The postoperative course was uneventful. Pulsation of the dura (due to CSF interaction with the defect of middle cranial fossa) resolved by 1 month after the operation. The defect of the middle cranial was closed by the epithelium at 2 months after the operation, and the pulsation of the dura had resolved by this time. Temporal bone computed tomography (CT) revealed no recurrence of the cholesterol granuloma at 4 years postoperatively (Figures 2(c) and 2(d)). Audiography showed that the preoperative hearing level of 56.3 dB (PDH) had improved to 33.8 dB2 years after the operation.

## 3. Discussion

Cholesterol granuloma of the middle ear typically presents with conductive hearing loss and a blue eardrum, and diagnosis is easy when these findings are present.

The present case did not display these characteristic findings (e.g., blue eardrum) but did show perforation in the ear membrane. Temporal bone CT and MRI were very helpful and allowed us to diagnose this disease. Temporal bone CT showed a soft-tissue mass that had destroyed the bony plate of the posterior and middle cranial fossa. MRI was performed to help differentiate between the various possible diagnoses (e.g., dermoid cyst, cholesteatoma, other tumors, and carotid aneurysm) [5]. MRI findings of this case revealed high signal intensity on T1-weighted images and a hyperintense periphery and hypointense interior on T2-weighted imaging. Cholesterol granuloma is characterized by high signal intensity on both T1- and T2-weighted images, due to the paramagnetic effects of protein content and cholesterol [6] and because of the presence of areas of void. In contrast, cholesteatomas appear to be iso- or hypodense on T1-weighted imaging and display high signal intensity on T2-weighted imaging [7].

A hyperintense appearance on T1- and T2-weighted imaging is a unique feature of cholesterol granuloma. The internal mass of the cholesterol granuloma displayed low signal intensity on T2-weighted imaging, attributed to bleeding within the cholesterol granuloma.

Furthermore, we performed DWI to differentiate cholesterol granulomas from cholesteatomas, which exhibit high-intensity signals on DWI. Cholesteatoma is a structure that contains keratin in the epithelium. This keratin is very muddy and limits the diffusion of water, resulting in high signal intensity on DWI [8]. In the present case, the lesion was not as hyperintense as that seen with cholesteatoma, leading us to suspect the presence of cholesterol granuloma.

Nager [9] described the pathogenesis of cholesterol granulomas as being dependent upon three factors: hemorrhage, obstruction of drainage, and impairment of ventilation. The source of blood may be infection, trauma, or inflammation, and obstruction to drainage coupled with lack of ventilation leads to stasis of erythrocytes and other tissue elements that break down to deposit cholesterol and other lipids. These irritants stimulate a foreign body reaction that leads to the formation of the granuloma.

The reasons for the aggressive extension of cholesterol granuloma into the middle ear are unknown. However, Beegun and Bottrill [10] suggested intense bleeding as an essential component for both the initiation and maintenance of aggressive cholesterol granuloma. Processes that may contribute to such intensive bleeding include the following.

*(1) Vacuum Hypothesis*. Obstruction of the drainage pathway leads to the reabsorption of gas, promoting the development of a vacuum with resultant seepage of blood from mucosal vessels [10, 11]. 

*(2) Exposed Marrow*. The exposed marrow hypothesis put forth by Jackler and Cho suggests that exuberant pneumatization of the temporal bone exposes marrow-filled spaces of the petrous apex. The resulting coaptation of the marrow and mucosa results in a proclivity towards hemorrhage. Sustained hemorrhage from exposed marrow elements provides the engine responsible for progressive cyst expansion [10, 11].

*(3) Robust Blood Supply*. This theory suggests that a robust blood supply, such as that of the sigmoid sinus, carotid artery, or large epidural vein, is essential for the maintenance and promotion of bony destruction [10].

We believe that this aggressive cholesterol granuloma occurs due to several factors. In the surgery employed in the present case, obstruction of the pathway from attic to antrum was observed. This obstruction of drainage could potentially have caused a vacuum with resultant seepage of blood from mucosal vessels. In addition, intense bleeding was observed at the mastoid bone marrow. We therefore thought that temporal bone might be the source of bleeding. The exposed marrow theory of Jackler and Cho relates to cholesterol granuloma of petrous apex.

Whatever the underlying situation was, as a result of broadly similar mechanisms, the bone marrow of the mastoid became hypervascular, leading to the formation of cholesterol granuloma of the mastoid.

Martin et al. [1] suggested that benign and aggressive cholesterol granulomas are often macroscopically distinct. The commonly encountered cholesterol granulomas are fluid-like expanding masses that contain lipids and cholesterol crystals surrounded by a thin fibrous lining. The second type is the rare aggressive cholesterol granuloma surrounded by a thickened wall of fibrous tissue that is capable of eroding bone. Surgical findings in the present case included a very thick cyst wall of the cholesterol granuloma. Thus, this case could be regarded as an aggressive type of cholesterol granuloma.

The suitability of surgical intervention depends on the location and size of cholesterol granuloma, the existence of symptoms, and diagnostic confirmation [12, 13]. Treatment strategies for cholesterol granuloma of the petrous apex include surgical drainage and excision of the capsule or drainage of the cyst [14–16]. In cholesterol granuloma of the mastoid, simple mastoidectomy with insertion of a ventilation tube or with additional mastoid obliteration is preferred. We were able to completely remove the cholesterol granuloma including the sclerotic region in the epitympanic cavity macroscopically by performing canal wall down mastoidectomy. Four years after surgery, no recurrence of cholesterol granuloma has been seen in the mastoid. However, follow-up observation is necessary in the future.

## 4. Conclusions

We have reported herein a case involving a large, aggressive cholesterol granuloma of the middle ear that eroded the middle cranial fossa. Cholesterol granuloma of the mastoid may cause various complications, such as destruction of the inner ear, inner ear fistula, and cerebrospinal fistula, in addition to progression in the cranium, and early detection and treatment are crucial. The best method of diagnostic evaluation is clinical examination and MRI. Complete removal of cholesterol granuloma is necessary for treatment of cholesterol granuloma of the mastoid.
